# Functionality screening to help design effective materials for radioiodine abatement

**DOI:** 10.3389/fchem.2022.997147

**Published:** 2022-10-18

**Authors:** Thomas J. Robshaw, Joshua Turner, Olivia Tuck, Caroline Pyke, Sarah Kearney, Marco Simoni, Clint A. Sharrad, Brant Walkley, Mark D. Ogden

**Affiliations:** ^1^ Department of Chemical and Biological Engineering, the University of Sheffield, Sheffield, United Kingdom; ^2^ Faculty of Life Sciences, University of Bradford, Bradford, United Kingdom; ^3^ National Nuclear Laboratory, Central Laboratory, Sellafield, Seascale, United Kingdom; ^4^ Department of Chemical Engineering and Analytical Science, the University of Manchester, Manchester, United Kingdom

**Keywords:** functionality, selectivity, radioiodine, adsorption, metal-loaded resin, screening, experimental design

## Abstract

This paper is part of a growing body of research work looking at the synthesis of an optimal adsorbent for the capture and containment of aqueous radioiodine from nuclear fuel reprocessing waste. 32 metalated commercial ion exchange resins were subjected to a two-tier screening assessment for their capabilities in the uptake of iodide from aqueous solutions. The first stage determined that there was appreciable iodide capacity across the adsorbent range (12–220 mg·g^−1^). Candidates with loading capacities above 40 mg·g^−1^ were progressed to the second stage of testing, which was a fractional factorial experimental approach. The different adsorbents were treated as discrete variables and concentrations of iodide, co-contaminants and protons (pH) as continuous variables. This gave rise to a range of extreme conditions, which were representative of the industrial challenges of radioiodine abatement. Results were fitted to linear regression models, both for the whole dataset (*R* ^2^ = 59%) and for individual materials (*R* ^2^ = 18–82%). The overall model determined that iodide concentration, nitrate concentration, pH and interactions between these factors had significant influences on the uptake. From these results, the top six materials were selected for project progression, with others discounted due to either poor uptake or noticeable iodide salt precipitation behaviour. These candidates exhibited reasonable iodide uptake in most experimental conditions (average of >20 mg·g^−1^ hydrated mass), comparing favourably with literature values for metallated adsorbents. Ag-loaded Purolite S914 (thiourea functionality) was the overall best-performing material, although some salt precipitation was observed in basic conditions. Matrix effects not withstanding it is recommended that metalated thiourea, bispicolylamine, and aminomethylphosphonic acid functionalized silicas warrant further exploration.

## Introduction

**FIGURE 1:**
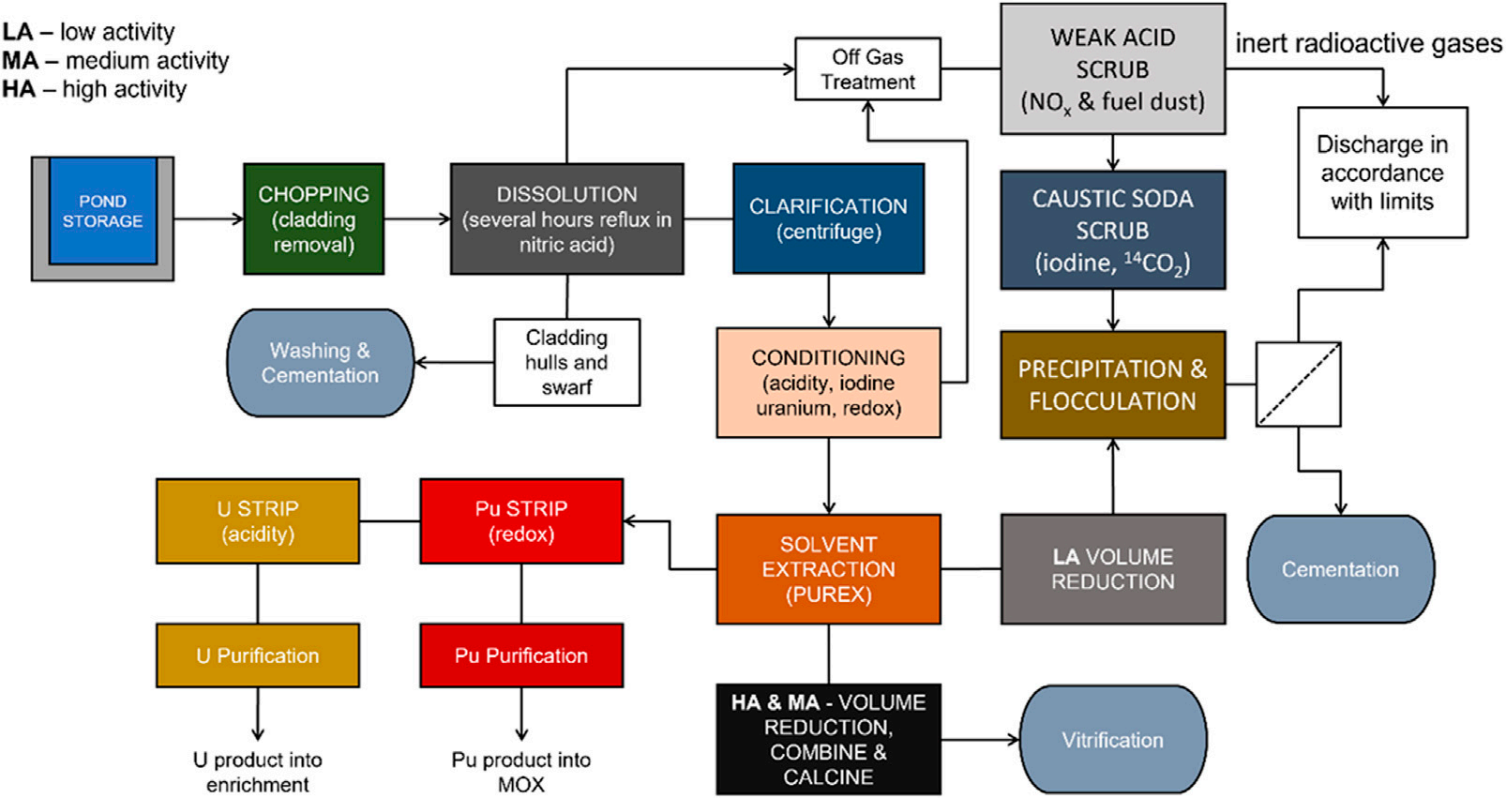
Generic UNF reprocessing scheme for a partially closed nuclear fuel cycle based upon an early split Plutonium and Uranium Redox Extraction (PUREX) separation and a simplified back end of Thermal Oxide Reprocessing Plant (THORP) (Wilson, 1996).

In current and proposed advanced nuclear fuel reprocessing schemes, the removal and abatement of residual radioiodine is particularly noteworthy. Typically 94–99% of the total iodine fission product content in used nuclear fuel (UNF) is volatilized during dissolution in nitric acid and enters the dissolver off gas stream (Riley et al., 2016), where it is captured by wet caustic scrubbing or adsorption on to a solid phase material (Figure 1). Gas-phase iodine absorption has been extensively studied (Haefner and Tranter, 2007; Agency, 2014; Huve et al., 2018) and a number of industrial systems are in full-scale operation (Riley et al., 2016).

There is however no effective method for the removal of the remaining fraction of iodine in the aqueous phase. In UNF reprocessing, the dissolver raffinate travels through various separation stages, with further iodine releases into the off-gas streams (Robshaw et al., 2021a). Therefore, an aqueous iodine-removal step could hypothetically be inserted at many stages of unit operations. Additionally, the aqueous iodine scrubbed from off-gas streams also requires appropriate remediation. Current practice involves controlled discharge into the ocean (Raisbeck and Yiou, 1999). While the process is performed within allowed limits for environmental release, it is ultimately unsustainable, given the sector-wide drive towards “near zero” release of radionuclides into the environment (Agency, 2018). Accordingly, current research trends are now focusing more on concentrating the iodine within an inert wasteform, suitable for storage within a deep geological facility (DGF) (Riley et al., 2015; Matyas et al., 2016; Maddrell et al., 2019).

Although the target iodine species in the target wastestreams are mainly anionic (Barton et al., 2019), the aim of selective remediation *via* conventional adsorption means is difficult. Whilst an anion-exchange mechanism appears to be the most plausible means of removal, some aqueous liquors within a UNF recycling plant can contain up to 9 M nitric acid, meaning there are generally macro concentrations of nitrate (and sometimes nitrite) in the liquors to be treated (Decamp and Happel, 2013; Asmussen et al., 2016). Another major source of competition could be the chelating oxo-anion molybdate (Mo-99), which does not volatilize to the same degree as iodine upon fuel dissolution (Zhirnov et al., 1997).

An effective adsorbent for aqueous radioiodine must be robust enough to (at least temporarily) withstand harsh chemical and radiolytic conditions, selective enough to retain appreciable iodine capacity in the presence of competitor species and be amenable to conversion to a stable final wasteform. A number of materials have been researched in recent years for this remit, which have been recently reviewed (Robshaw et al., 2021a). The huge majority incorporate iodine-selective metals into the adsorbent to create the required selectivity, with the most popular metals being Ag, Bi, Cu and Pb (Kodama, 1999; Lefevre et al., 1999; Zhang et al., 2011; Al-Mamoori et al., 2020). Candidates for the bulk phase of the adsorbent have included silicates (Asmussen et al., 2019; Cordova et al., 2020), titanates (Bo et al., 2013; Liu et al., 2015) and metal oxides/hydroxides (Mao et al., 2016; Chen et al., 2018). The issue with many such adsorbents is that the active species, to be converted into a stable iodide salt, tend to be somewhat stable themselves and moreover, present in the form of nanoparticles on the adsorbent surface. This entails a slow oxidation/dissolution/precipitation uptake process, which causes the kinetics to be hindered (Kodama, 1999; Tauanov and Inglezakis, 2019) and means that not all of the valuable metal centers are being used effectively for capture.

**FIGURE 2:**
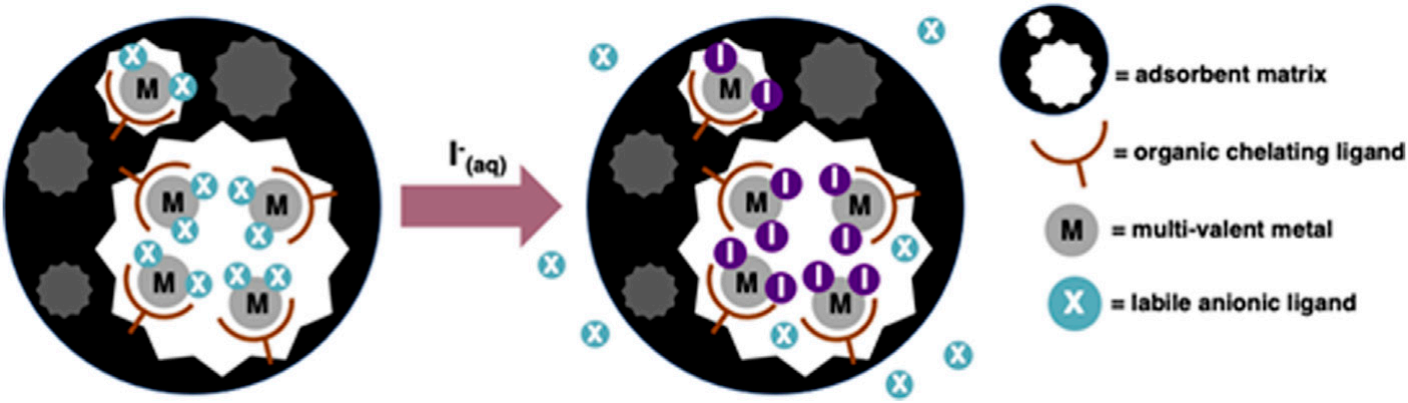
Demonstration of the uptake mechanism for a metallated adsorbent, operating *via* complex-formation and ligand-exchange.

In contrast, a hybrid adsorbent can function *via* the grafting of organic ligands to the skeleton of the material. Each ligand then coordinates a selective metal ion, which in turn ligates the iodine from solution as iodide (Robshaw et al., 2019a; Robshaw et al., 2020) (Figure 2). Materials operating *via* ligand-exchange reactions often exhibit fast kinetics (Blumberger et al., 2004; Robshaw et al., 2019b) and possibly improved selectivity, depending on variations in thermodynamic stability between the formed iodide complex and contaminant complex. Furthermore, provided there is adequate transport of ions within the adsorbent pores, every loaded metal ion can theoretically be used to bind iodide ligands (Robshaw et al., 2020).

Commercial polymeric ion-exchange resins offer facile access to a large library of chelating ligands, with proven metal-capture capabilities and are therefore ideal for adsorbent screening processes (Riley et al., 2018; Bezzina et al., 2019). However, once such materials are loaded with the selected metal ions, the functionalities then essentially become novel in nature and there are many unknowns regarding how the adsorbent will then behave in an anion-capture remit. The mechanisms of uptake are often unclear and unexpected when characterized (Millar et al., 2017; Robshaw et al., 2019a). Furthermore, if the attraction between metal and anion is far in excess of that between metal and adsorbent ligand, the formation of salts *in situ* can occur (Millar et al., 2017; Robshaw et al., 2019c), which could be very problematic in the design of a hydrodynamic process.

In conventional research into the application of a novel adsorbent for a certain remit, the material is often subjected to a number of experiments to assess its suitability for the task. These include the generation of isotherms, to derive a maximal capacity (Zhang et al., 2019), a pH profile, to determine at what level of acidity or alkalinity the uptake mechanism breaks down (Bezzina et al., 2018; Robshaw et al., 2019c) and tests against various concentrations of co-contaminants, to investigate suppression of the ion of interest by other species. These tests, while generating valuable physicochemical data, are time-consuming and in this instance, both a large number of candidate ligands and four potential functionalizing metals warrant investigation. There are obvious practical benefits to reducing the number of laboratory experimental steps required to make a robust decision in delivering an engineering solution; not least the facts that costs, energy use and environmental impact of the work is minimized.

This study therefore utilizes statistical experimental design methods to rapidly down-select a wide range of candidate adsorbents. This minimizes the number of experimental steps required to produce a shortlist of “champion” candidates, which can then be subjected to more rigorous testing.”. From a pool of 32 materials, six candidates are identified as meriting further investigation, which is achieved over just two screening experiments and aided by employing fractional factorial experimental design. Furthermore, the experiments assist in identifying the aqueous conditions, favourable and unfavourable to iodine removal; helping to inform where the potential iodine-removal step is placed, with respect to spent nuclear fuel reprocessing unit operations.

## Experimental

### Chemical reagents

Eight commercial ion-exchange resins were acquired for these experiments by kind donation from the manufacturers and are displayed in Table 1. Ion-exchange resins, as-received, are generally supplied in partially protonated or deprotonated (sodium) form, with a variety of counter-anions. For uniformity, prior to metallation, all resins were immersed in 1 M HNO_3_ (∼25 g as-received mass in 1 L) and pre-conditioned in an orbital shaker for a minimum of 24 h. The resins were then rinsed with 5 L of deionized water and stored in deionized water until required.

**TABLE 1:** Overview of basic physicochemical properties of the resins used in these experiments.

Commercial name	Functional group	Quoted exchange capacity (mol·L^−1^)	Moisture retention, manufacturer-quoted (%)	Common industrial uses
Ambersep M4195	Bispicolylamine (BPA)	0.52	40–60	Selective Cu and Ni extraction from acidic solutions
Purolite MTS9301	Iminodiacetate (IDA)	1.57	52–62	Co., Cu, Ni and Zn extraction from acidic solutions
Puromet MTS9140	Thiourea	1.0	50–56	Ag and Au removal from pregnant leach solutions
Puromet MTS9850	Polyamine	2.3	52–57	Removal of heavy metals, present as anionic complexes in neutral to mildly alkaline solutions
Lewatit A365	Polyamine/amide	1.1	44–51	Demineralisation of high TDS solutions. Selective sulfate removal
Puromet MTS9501	Aminomethyl-phosphonic acid (AMP)	0.60	60–68	Removal of heavy metals at low pH. Removal of alkali earth metals at high pH
Puromet MTS9100	Amidoxime	1.90	52–60	Cu and Fe removal at low pH. Precious metal extraction from dilute solutions
Purolite CT275	Sulfonic acid	2.86	51–59	Demineralisation for production of fine chemicals

It can be seen that most of the chosen resins are quoted by the manufacturers as being suitable for the capture and immobilization of one or more of the previously-mentioned iodine-selective metals. The exceptions were Lewatit A365, which has previously been investigated for iodine remediation (Barton et al., 2019). It was predicted that the mixture of amine and amide functionality might produce metal chelating properties that were similar, but not identical to Purolite MTS9850. Purolite CT275 was selected with a view to demonstrate the effectiveness of a very economical non-chelating product, in comparison to the more expensive bespoke functionalities tested.

Solutions of the required metal ions were made by dissolution of the appropriate salts. AgNO_3_ (Sigma Aldrich), BiCl_3_ (Flurochem), CuCl_2_.2H_2_O (Sigma Aldrich) and Pb(NO_3_)_2_ (Fisher Scientific) were all analytical grade and were dissolved in the appropriate volume of deionized water to make 0.10 M solutions, with the exception of BiCl_3_, which hydrolyses slowly in water. This was dissolved in anhydrous acetone (Fisher Scientific).

Solutions of iodide anions, with controlled amounts of other species added, were made up by dissolving the appropriate salts in deionized water (all analytical grade). Sodium iodide, sodium chloride, sodium molybdate and sodium sulfate were all purchased from Sigma Aldrich. The pH of solutions was adjusted by addition of HNO_3_ or NaOH and final solution pH was determined either by potentiometric titration against standard solutions, or by measurements with a calibrated pH electrode.

### Metallation of ion-exchange resins

The chosen resins were loaded with metal ions as follows. ∼0.5 g of resin (hydrated mass) was placed in a polypropylene Digisep tube and 50 ml of 0.10 M metal ion solution was added. The tube was sealed and placed on an orbital shaker for 48 h, to allow equilibrium to be reached. The metallated resins were rinsed with 500 ml of deionized water (or acetone, in the case of Bi-loaded materials) and stored in deionized water until required.

### Iodide uptake experiments from aqueous solutions

For determination of iodide uptake from NaI solutions, ∼0.1 g of metallated resin (hydrated mass) was placed in a polypropylene Digisep tube and 25 ml of solution was added, with an iodide concentration of 1.00 g L^−1^. The tube was sealed and placed on an orbital shaker for 48 h, to allow equilibrium to be reached. 2.5 ml aliquots were removed from the equilibrated solutions and transferred to further Digisep tubes. To these were added 500 μL of 5 M NaNO_3_ buffer solution and the samples were made up to a final volume of 25 ml in preparation for electrode analysis.

For determination of iodide uptake from solutions with various conditions and levels of other contaminants, ∼50 mg of metallated resin (hydrated mass) was placed in a polypropylene Corning centrifuge tube and 10 ml of solution was added. The tube was sealed and placed on an orbital shaker for 48 h, to allow equilibrium to be reached. 2.5 ml aliquots were removed from the equilibrated solutions and transferred to Digisep tubes. For samples of low pH (0), 2.5 ml of 1 M NaOH was added, to neutralize the solution. For all samples, 500 μl of 5 M NaNO_3_ buffer solution was then added, and the samples were made up to a final volume of 25 ml in preparation for electrode analysis. The pH change during the adsorption equilibrium process was not monitored. Previous work indicated that any changes would be negligible (Robshaw et al., 2020).

### Iodide quantification by ion-selective electrode (ISE)

Determination of iodide concentration in sample solutions was performed using a Cole Parmer iodide selective electrode. This was calibrated, prior to analysis and after a maximum of 20 measurements, using standards of 0.02–2 or 2–200 mg L^−1^, made up from a 1,000 (±3) mg·L^−1^ stock solution from Cole Parmer. Three electrode measurements were taken per sample. Iodide uptake of the metallated resins, under various conditions, was calculated by mass-balancing, using Eq. 1:

$$q_{e}=\left(C_{1}-C_{2}\right)V/m$$ (1)

where q_e_ is the equilibrium iodide uptake of the adsorbent (mg·g^−1^, hydrated mass basis), C_1_ is the iodide concentration in the solution before contact with the adsorbent (mg·L^−1^), C_2_ is the iodide concentration in the solution after contact, at equilibrium, V is the volume of solution treated (L) and m is the hydrated mass of the adsorbent. All samples were analyzed in duplicate and average values for the adsorbent performance were calculated (Maimaitiniyazi and Zhou, 2022). The associated errors with each individual q_e_ values were calculated using 2 x standard deviation over the three electrode measurements for each sample. Total error values were then propagated, using standard methods.

The iodide distribution coefficient (K_D_) for each material was calculated using Eq. 2:

$$K_{D}={\left[I^{-}\right]}_{\left(org\right)}/{\left[I^{-}\right]}_{\left(aq\right)}$$ (2)

Where [I^−^]_(org)_ is the concentration of iodide in the organic phase (adsorbed on to the resin) at equilibrium (mg·kg^−1^) and [I^−^]_(aq)_ is the concentration of iodide in the aqueous phase at equilibrium (mg·L^−1^) (Everett, 1998). K_D_ is more commonly employed in solvent extraction experiments but is useful when investigating the affinity of an adsorbent for a certain adsorbate, when the initial adsorbate concentration varies throughout experiments, as it does in this case.

### Batch iodide uptake from simple aqueous solution (the first experimental round)

In the initial experimental round, all 32 of the synthesized materials were assessed for their iodide uptake capabilities from analytical NaI solutions only, using the conditions previously described. Following analysis of these initial results, materials which exhibited an iodide capacity of ≥40 mg g^−1^ were progressed to the second round. All others were discarded and not assessed further.

### Fractional factorial experimental design (the second experimental round)

Each material assessed was explored individually using a fractional factorial design for all key exploratory variables. The key exploratory variables are all continuous (six in total). These include iodide concentration, pH, nitrate concentration, chloride concentration, sulfate concentration and molybdate concentration. A 2_IV_^6-2^ design specification was chosen (Box et al., 1978). This required a total of 19 different contact solutions to be made up, as per 2.1. This included 16 contact solutions with various combinations of continuous variables at designated “high” and “low” levels. To estimate variability in the experimental method and material properties, as well as establishing curvature in the design space, repeated contact solutions were produced at a “centre-point”. For these “centre-points”, the continuous variable values were an exact mid-point between “high” and “low”. A breakdown of the composition of each contact solution is shown in Table 2. The high and low values for the continuous exploratory variables were determined either by known limitations of the adsorbents (For example, the inorganic matrix forms of a number of these adsorbents are known to dissolve at pH > 12 (Lambert, 2020)) or by the ranges reported in the literature (Robshaw et al., 2021a). It should be noted that Table 2 is only a representation of the experimental design and we do not claim the values are accurate with respect to the actual measured concentration of each analyte. In other words, the table does not account for changes in speciation that would occur as the solutions were mixed and the different components reached equilibrium. This is especially significant in terms of the iodine speciation and will be discussed later.

**TABLE 2:** Chemical composition of the 19 contact solutions made up for the fractional factorial experiment. Red = “low” value of variable. Green = “high” value of variable. Yellow = centrepoint value of variable. Orange = non-ideal concentration, due to essential addition to create acidity.

Solution References	[Iodide] (mg·L^−1^)	pH	[nitrate] (mg·L^−1^)	[chloride] (mg·L^−1^)	[molybdate] (mg·L^−1^)	[sulfate] (mg·L^−1^)
A	10	0	62,000	0	0	0
B	1,000	0	62,000	0	1,600	0
C	10	12	0	0	1,600	2,342
D	1,000	12	0	0	0	2,342
E	10	0	248,000	0	1,600	2,342
F	1,000	0	248,000	0	0	2,342
G	10	12	248,000	0	0	0
H	1,000	12	248,000	0	1,600	0
I	10	0	62,000	852	0	2,342
J	1,000	0	62,000	852	1,600	2,342
K	10	12	0	852	1,600	0
L	1,000	12	0	852	0	0
M	10	0	248,000	852	1,600	0
N	1,000	0	248,000	852	0	0
O	10	12	248,000	852	0	2,342
P	1,000	12	248,000	852	1,600	2,342
Q	505	6	124,000	426	800	1,171
R	505	6	124,000	426	800	1,171
S	505	6	124,000	426	800	1,171

It can be seen from Table 2 that the actual nitrate values did not exactly follow the experimental design. This was because nitrate was necessarily added in the form of HNO_3_ to reduce pH. As such, nitrate and pH factors are not completely independent. This complication was unavoidable because, as per Table 2, chloride and sulfate were also continuous variables; so it was not possible to reduce pH effectively as required without making some adjustment away from the ideal experimental design. The decision to use HNO_3_ for pH adjustment was essentially because the concentration of nitrate generated by adjusting the acidity was still significantly below the centre-point matrices; whereas if HCl or H_2_SO_4_ had been used, the resulting chloride or sulfate concentration for those solutions would have been above the designated “high” values for each variable.

The total number of samples analyzed in the fractional factorial round was dictated by the number of materials progressed from the first experimental round (19 × 2 duplicate reps = 38) and the number of contact solutions required by the design specification (19). This resulted in 19 × 38 = 722 samples. The order of electrode analysis of these 722 samples was randomized, using a Microsoft Excel function. The samples were analyzed in six sessions of approximately 3 h, over 3 days, with electrode recalibrations as previously stated.

### Regression modelling

Linear regression was applied in the computer programme “R” (Team, 2018) to find relationships between the continuous exploratory variables and the outcome variable(s). The models were used to make predictions over the chemical regions tested to help identify optimal regions for future work.

Linear regression models take the form:

$$y_{i}=\beta_{0}+\beta_{1}∙x_{i1}+\ldots +\beta_{n}x_{in}+\varepsilon_{i}$$ (3)

where y_i_ is the dependent variable, the x_n_ represent the n independent variables, the βn are parameters to be estimated and ε_i_ is the random error (described by a normal distribution with a mean of zero and a standard deviation determined by distance of the residuals to the regression line ∼ $N\left(0,\sigma^{2}\right)$ ). Transformations, generally on the dependent variable, may be required on the modelled data to ensure the model assumptions are met. For a combined model incorporating all material types (a discrete variable), the linear models work by setting one level (one material type) as the baseline and giving the change from that baseline for the other levels in the form of a constant added to Eq. 3, specific to the discrete parameter. For this work, this β parameter was termed the “material offset value”.

For a $2_{IV}^{\;6-2}$ fractional factorial design two-way interactions are confounded with certain other two-way interactions. As such, a forward step-wise regression approach was used to first identify the significant main effects, and then include the two-way interactions between the significant main effects only.

Linear regression models were checked using [A] *R* ^2^, which can be understood as a percentage of variation in the data explained by the model; [B] Residual plots, used to understand the goodness of fit for each model. Residuals (the remaining random error, ε_i_) are the difference between each data point and the fitted model. Four plots are presented which are: [i] Residuals vs. fitted values. Plotted against fitted value, a good model will show a random scatter around 0 and no obvious trend, i.e. increasing/decreasing or curvature. [ii] Normal Q-Q. This is a check for normality of the residuals. The dashed line shows an exactly normal distribution but in real data some variation is expected, particularly in the tails. [iii] Scale-location. This plots the square root of residuals (absolute values) against fitted values. As with residuals against fitted value, there should be no trends in the scale-location plot. [iv] Residuals vs. Leverage. This plots the standardized residuals against the cook’s distance which is a measure of Leverage. This indicates whether an individual point has excessive influence over the model fit, potentially lying separate from the main body of points at the tail of the distribution of y and yet having a strong effect on the gradient.

## Results and discussion

### Batch iodide uptake from simple aqueous solution (the first experimental round)

**FIGURE 3:**
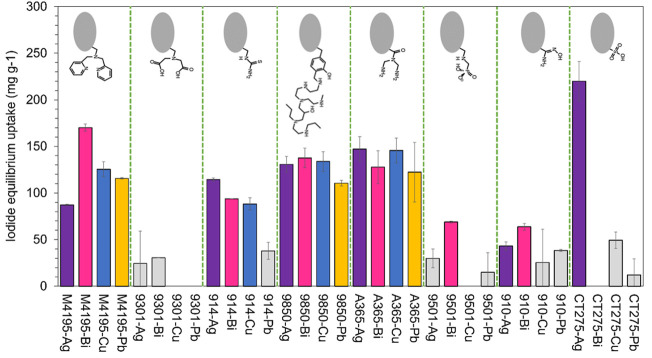
Uptake of iodide from NaI solutions by the 32 different metallated adsorbents. Iodide concentration = 1.00 g L^−1^. Solution volume = 25 ml. Adsorbent hydrated mass ≈0.1 g. Contact time = 48 h. T = 20°C. Materials not progressed to second round are shown in grey.

This round was instigated with the intention of effecting a down-selection process using the simplest possible experimental setup and an uncomplicated results analysis. This was in order to reduce the number of candidate materials (and therefore the number of experiments and analyses) that would be processed in the fractional factorial round. The fractional factorial approach greatly reduces the total number of samples that must be produced and analyzed, in order to derive which continuous variables are significant to adsorbent performance. However, the approach is still rigorous and time-consuming in terms of the number of laboratory and data-processing hours required for the 722 samples. Therefore, the purpose of this initial round was to “scope” all 32 adsorbents for their ability to remove iodide from analytical NaI solutions, essentially awarding each metallated resin with a “go/no go” status, with respect to appreciable capacity. The iodide capacity achieved by every assessed material in the first screening experiment and progression status after the first round are shown in Figure 3 (more detail can be seen in Supplementary Figure S1).

Overall, The Ag-loaded materials possessed the highest mean capacity of 99.6 mg g^−1^. This however was heavily skewed by the very high uptake of CT275-Ag. The mean capacities for the other metals were in order 86.6 mg g^−1^ (Bi), 71.0 mg g^−1^ (Cu) and 51.1 mg g^−1^ (Pb). The resins exhibiting the greatest mean capacity, considering all metallated forms, were A365 (136 mg g^−1^), MTS9850 (128 mg g^−1^) and M4195 (125 mg g^−1^). S914 had a reasonable mean capacity of 83.7 mg g^−1^. The CT275 mean capacity was 70.4 mg g^−1^ (again, strongly influenced by the CT275-Ag result). The lowest-performing materials in this regard were S910 (42.6 mg g^−1^), MTS9501 (28.4 mg g^−1^) and MTS9301 (13.8 mg g^−1^). The order of capacities is likely partially due to the fact that the amine groups in the most capacious resins would have been mainly protonated in the experimental conditions used (Ogden et al., 2017; Barton et al., 2019) and therefore act as anion-exchange sites, in addition to the intended metal/iodide interaction.

Comparing Table 1 with Figure 3, it can be seen that the iodide capacity of a given resin does not directly correlate to its manufacturer-quoted exchange capacity. There are two likely reasons for this. The first is that the metal ions chosen are multivalent (including Ag, despite the ions only being singly-charged) and the binding of metals on to most of these resins does not take place by the linear exchange of one proton for one metal ion (Pepper et al., 2018; Bezzina et al., 2019; Robshaw et al., 2020). However, the exchange capacity of ion-exchange resins is routinely done by quantifying the exchange of sodium ions for protons, by back-titration (Fisher and Kunin, 1955). The second reason is the issue of the formal charge of each respective ligand. For example, the BPA group is a neutral ligand (Robshaw et al., 2020) and it follows that a 2 + cation coordinated to a BPA group may further coordinate two anionic ligands to form a charge-neutral complex, whereas a 3 + cation may coordinate three such ligands. This behaviour can be seen in Figure 3, with M4195-Bi having rather greater iodide uptake values than M4195-Cu and M4195-Pb, which in turn are greater than M4195-Ag. In contrast, the IDA functionality has a formal 2- charge (Bezzina et al., 2020), assuming metal ions are coordinated to both carboxylate groups simultaneously, with the exchange of protons (Table 1). Therefore, further coordination of anionic ligands is unfavourable and even though the metallic affinity for iodide is high, it does not counteract the favourability of the chelating interaction. MTS-9301 accordingly was one of the worst-performing materials tested.

The CT275 resin produced low uptake in conjunction with all metals except Ag. This is presumed to be because two or three sulfonic acid groups in close vicinity interact simultaneously with 2+ and 3 + cations (James et al., 2019), again meaning that coordination of anionic iodide ligands is unfavourable. The exception is Ag, which has such strong affinity for iodine species (Chen et al., 2018; Asmussen et al., 2019) that there is likely *in situ* formation of AgI within the resin pores. However, in simple NaI solutions, the crystals appear to be electrostatically anchored to the adsorbent surface (which, as shall be seen, was not always the case in the second experimental round). Such phenomena have been observed before in the case of lanthanum and fluoride interactions (Robshaw et al., 2019c).

From these results, a total of 19 materials were progressed to the second experimental stage. The arbitrary cut-off observed capacity of 40 mg g^−1^, to differentiate between “go” and “no go” materials, was essentially dependent on practicalities, as previously described. The materials which were not further studied are indicated in Figure 3. It should be noted that precipitation was observed in the case of the CT275-Cu samples, in the form of free salt particles in the contact solution and this is the reason the material was not progressed, rather than uptake capacity.

### Batch iodide uptake from various media, following fractional factorial experimental design (the second experimental round)

**FIGURE 4:**
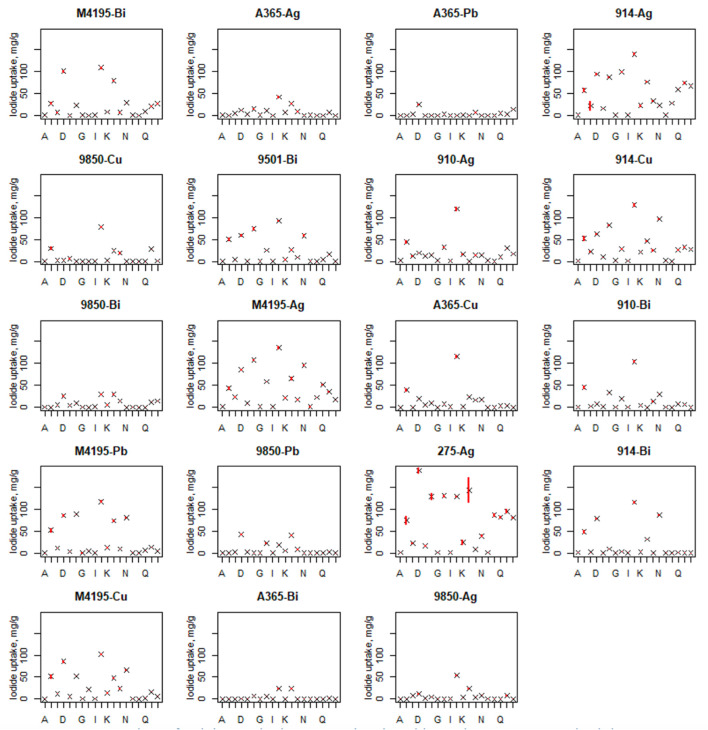
Showing the full experimental dataset for iodide adsorption by the candidate materials over all experimental conditions. Solution volume = 10 ml. Adsorbent hydrated mass ≈0.05 g. Contact time = 48 h. T = 20 °C. *X* axis lettering denotes the contact solution conditions outlined in Table 2.

A visualisation of the entire dataset attained for the second experimental round can be seen in the scatter plots shown in Figure 4 (boxplots for iodide uptake performance is shown in Supplementary Figure S2). This figure shows the large number of 0 uptake values that were recorded for many materials, in the challenging conditions. The full graphical representation of the analysis is found in the Supporting Information. Two of the best-performing materials, M4195-Ag and CT275-Ag, along with S910-Ag were observed to produce significant salt precipitation during contact with the majority of solutions tested. The most favourable conditions for iodide uptake for all materials were produced by solution J. The least quantity of iodide was generally taken up from solutions A, G, I and O, which all had a low initial iodide concentration.

### Data modelling *via* statistical analysis

The presence of 0 iodide uptake values in the dataset impacted the linear regression modelling (as well as suggesting that the relevant materials would not be suitable for iodide uptake in industrial conditions). Hence, materials with multiple 0 values, particularly when the centre-points were 0, were removed in the construction of this model. This issue could potentially be negated in the future by using a larger resin to solution ratio. However it did not interfere in the key experimental goal of identifying higher and lower performing adsorbents. Materials included and excluded in the model are given in Table 3.

**TABLE 3:** Showing the discreet variables for which, the data were considered in the construction of the overall group model.

Included	M4195-Bi, S914-Ag, MTS9501-Bi, S910-Ag, S914-Cu, M4195-Ag, S910-Bi, M4195-Pb, CT275-Ag, M4195-Cu
Excluded	A365-Ag, A365-Pb, MTS9850-Cu, MTS9850-Bi, A365-Cu, MTS9850-Pb, S914-Bi, A365-Bi, MTS9850-Ag

The model requires a fourth root transformation to meet the normality requirements for linear regression. The model, with data for the materials included in Table 3, found [iodide], pH and [nitrate] to be significant, including the two-way interactions between all three, while [chloride] [molybdate] and [sulfate] were not found to be significant. The fitted equation is:

uptake14=1.55+0.0019×[iodide]+0.069×pH+(2.41×10−6)×[nitrate]−(8.30×10−5)×[iodide]×pH−(2.40×10−9)×[iodide]×[nitrate]−(4.34×10−7)×pH×[nitrate]+material offset (4)

where the material offset is given in Table 4, as the change from the arbitrary base material CT275-Ag. The *R* ^2^ for this model is 59% which shows that over half of the variability in the data is explained by the model. Statistically, the material offset should be thought of as an empirical constant, which allows for superior model-fitting, but also represents the overall affinity of each material for iodide capture across all experimental conditions. The values in Table 4 can also be used to rank the materials in terms of overall iodide uptake across all experiments. 275-Ag has the overall highest iodide uptake and 910-Bi the lowest.

**TABLE 4:** The 10 candidate materials included in the group model, ranked in order of material offset. Table also includes the calculated mean iodide capacity across all sample solutions Grey cells indicate materials for which precipitation was observed across multiple experiments.

Material	Offset	Mean uptake (mg·g^−1^)
CT275-Ag*	0	64.0 ± 4.0
S914-Ag	−0.15	45.4 ± 1.7
M4195-Ag*	−0.25	42.7 ± 1.0
S914-Cu	−0.42	35.7 ± 1.0
M4195-Cu	−0.66	29.0 ± 0.8
M4195-Pb	−0.67	32.4 ± 0.7
S910-Ag*	−0.79	19.1 ± 0.6
MTS9501-Bi	−0.87	24.3 ± 0.7
M4195-Bi	−0.90	24.1 ± 0.5
S910-Bi	−1.11	15.7 ± 0.4

The residual *versus* fitted plots are shown in S3. The development of individual models is presented in S4. The residual vs. fitted plots generally shows a good random scatter. The straight line of points that decreases as fitted values increases are mostly data points with a 0 uptake measurement, hence the model does not fit these points well. This is the same for the scale-location plot. The q-q plot shows a good fit to the normal line, with the exception in the bottom tail for the same outliers as the residual plot. The residual vs. leverage plot does not highlight any influential points.

**FIGURE 5:**
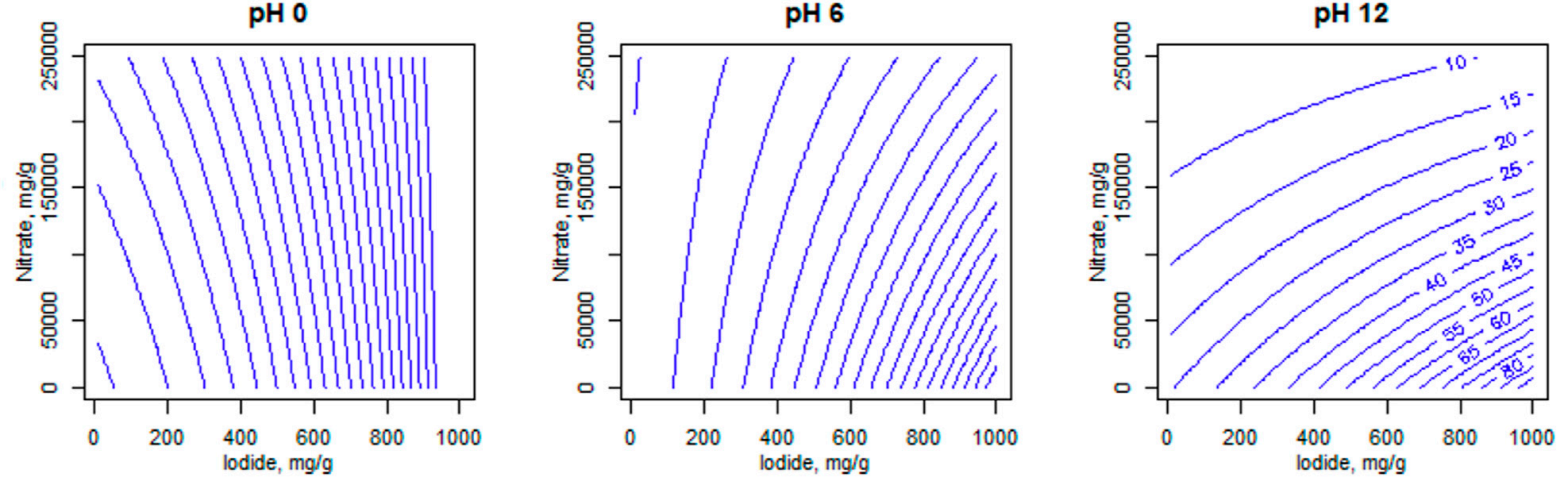
Predicted contour plots for S914-Ag at different solution pH values, based on the overall group model. Blue numerical values on contour lines represent predicted iodide uptake.

The fitted model can be used to make predictions across the range of input parameters and these are used to make contour plots to visualise the areas for best iodide uptake. These are shown for the 10 materials included in the model in Supplementary Figure S5-S14, with an example shown in Figure 5. Iodide and nitrate concentrations are varied continuously on the *x* and *y* axes respectively, while pH is varied at three levels, one for each plot, and predicted iodide uptake is shown in the contours in 5 mg g^−1^ intervals. This could be a useful predictor of how the uptake properties of an adsorbent can change in response to solution chemistry of the feed (if plant parameters are altered). For example, it can be seen that the suppressive effect of nitrate is rather greater at pH 12 than at acidic or neutral pHs (Supplementary Figure S5-S14). This is likely the result of a pH-induced speciation change from I^−^ to IO_3_^−^, which is known to have lower affinity for (at least) Ag (Asmussen et al., 2019), this being more affected by the nitrate competition. Hence, the contour plots are regarded as one of the most informative outcomes of the statistical analysis.

In general, it is noted that the significance of two-way interactions in the group model has shown the benefit of including all of the input parameters at this project stage and using a fractional factorial design to minimise the number of experiments required. If only one or two parameters were tested at a time, the interactions could have been missed and less informed decisions made for future work.

### Use of statistical analysis in the down selection process

Following the second experimental round, the decision was taken to use the required data analysis to reduce the number of candidate materials from 19 to 6. This figure was based on practicalities associated with the next experimental steps, which were expected to be time-consuming.

Because no decisions were made at this point as to which types of active aqueous waste should be targeted for radioiodine remediation, the basis of the down selection was the material offset value (Table 4). However, resins for which salt precipitation had been observed in more than two different sample solutions (Table 4) were also excluded, as the end goal technology for this work is a hydrodynamic system and a material likely to induce salt precipitation is inherently unsuitable for the remit (if the particle size of an ion-exchange column starts to change during operation, this influences the pressure required to pump the inlet solution through the adsorbent bed (Harland, 1994)). Thus, the six functionalities chosen to progress to further trials were M4195-Bi, M4195-Cu, M4195-Pb, S914-Ag, S914-Cu and MTS9501-Bi.

**FIGURE 6:**
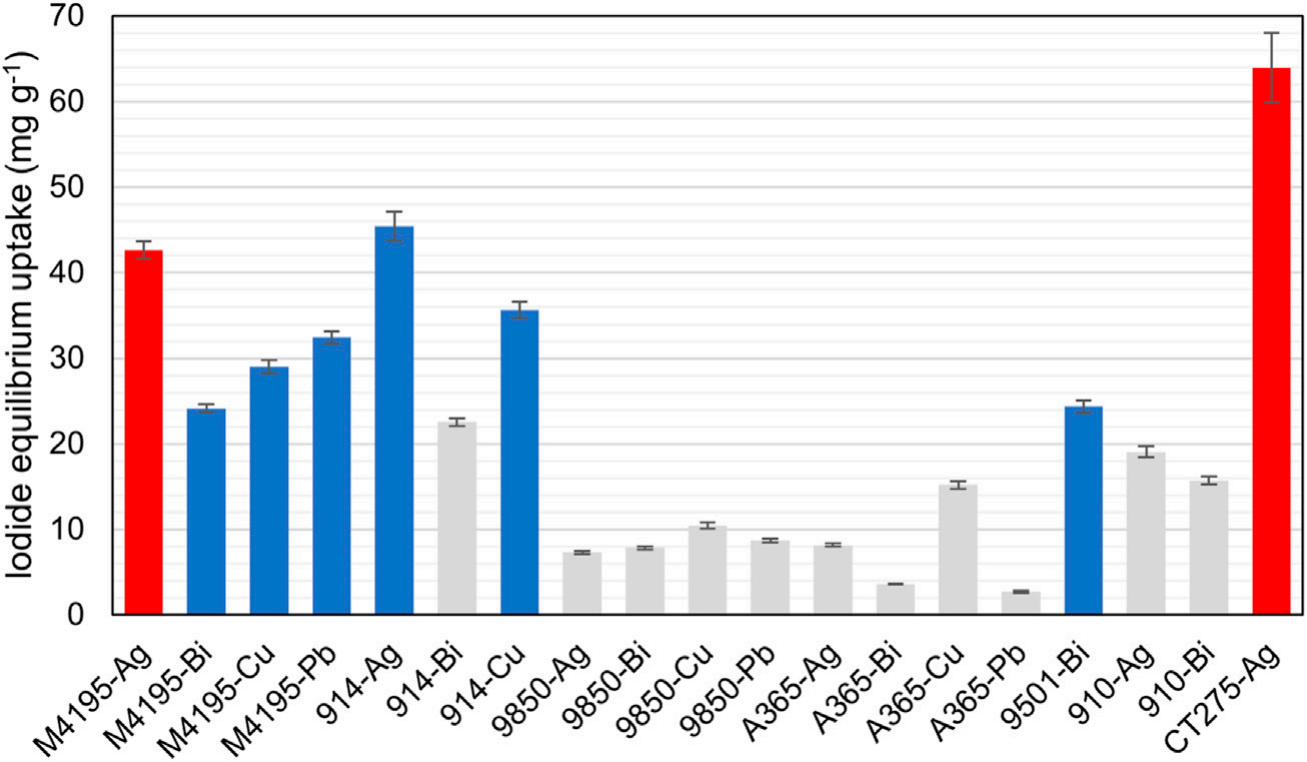
Showing the mean iodide uptake capacity of the 19 materials progressed to the fractional factorial experiment. Parameters are as per Figure 4. Red bars = not progressing due to observed precipitation. Grey bars = not progressing due to insufficient iodide uptake. Blue bars = progressing.

As a simple additional measure, we also calculated a mean iodide uptake for each material across all experiments. For this purpose, the mean value of centre-point samples was taken for each material, then an overall mean uptake value for that adsorbent was calculated, using values from solutions A-P and the single value calculated for solutions Q, R, and S. In other words, the centre-point uptake value was not given higher weighting in the calculation than the value for any other set of conditions. Notably (and again disregarding precipitation observations), the top six ranked candidates according to this criterion were exactly the same as those ranked by using the model-generated offset value (Figure 6).

### Influence of industrially realistic parameters on adsorbent uptake performance

The data from the second experimental round was, for many resins, a complete reversal of fortunes and shows the importance of simulating industrially realistic parameters when selecting a suitable adsorption media. For example, both MTS9850 and A365 performed very poorly in most conditions, despite clearly having high theoretical iodide uptake capacity, seen in the first experiment. This can be partially explained in that, as previously mentioned, the majority of iodide uptake by MTS9850 and A365 in the first round was thought to be *via* anion-exchange at protonated ammonium sites; these being far less selective than the metal centres. Although iodide is fairly high in the classical order of anion selectivity for weak/strong base functionality (Helfferich, 1962), the macro concentrations of nitrate and significant levels of molybdate, chloride and sulfate have all been observed to have a suppressive effect on iodide capture by these resins in the past (Barton et al., 2019; Robshaw et al., 2020). This does not however, explain why the metal centres themselves also appear to be deactivated to iodide uptake. It is possible that these two functional groups are less resistant to competitive binding from other anions because of the steric and geometric restrictions of the binding environments.

The second round illustrated the importance of a strong chelating interaction between functional group and metal ion, to prevent deleterious precipitation processes occurring. S914 was one of the strongest performing resins in the second round. It exhibited only middling iodide capacity in the first round, due to its relatively low degree of functionalisation (Table 1), but it can be seen that the ligand/metal combinations formed appear to be highly stable under challenging conditions. This can be partially attributed to irreversible REDOX reactions in the case of Cu (Bezzina et al., 2020). M4195 was high performing in both rounds. A number of literature studies have noted strong binding interactions with the loading metals, attributed to interactions with the multiple nitrogen atoms (Diniz et al., 2002; Ogden et al., 2017). However, in the case of Pb, the useful performance in the second round was unexpected, as Pb^2+^ ions are known to be too large to fit into the bispicolylamine ‘cavity’; thus a weaker ligand-metal interaction would be predicted (Bezzina et al., 2020).

**FIGURE 7:**
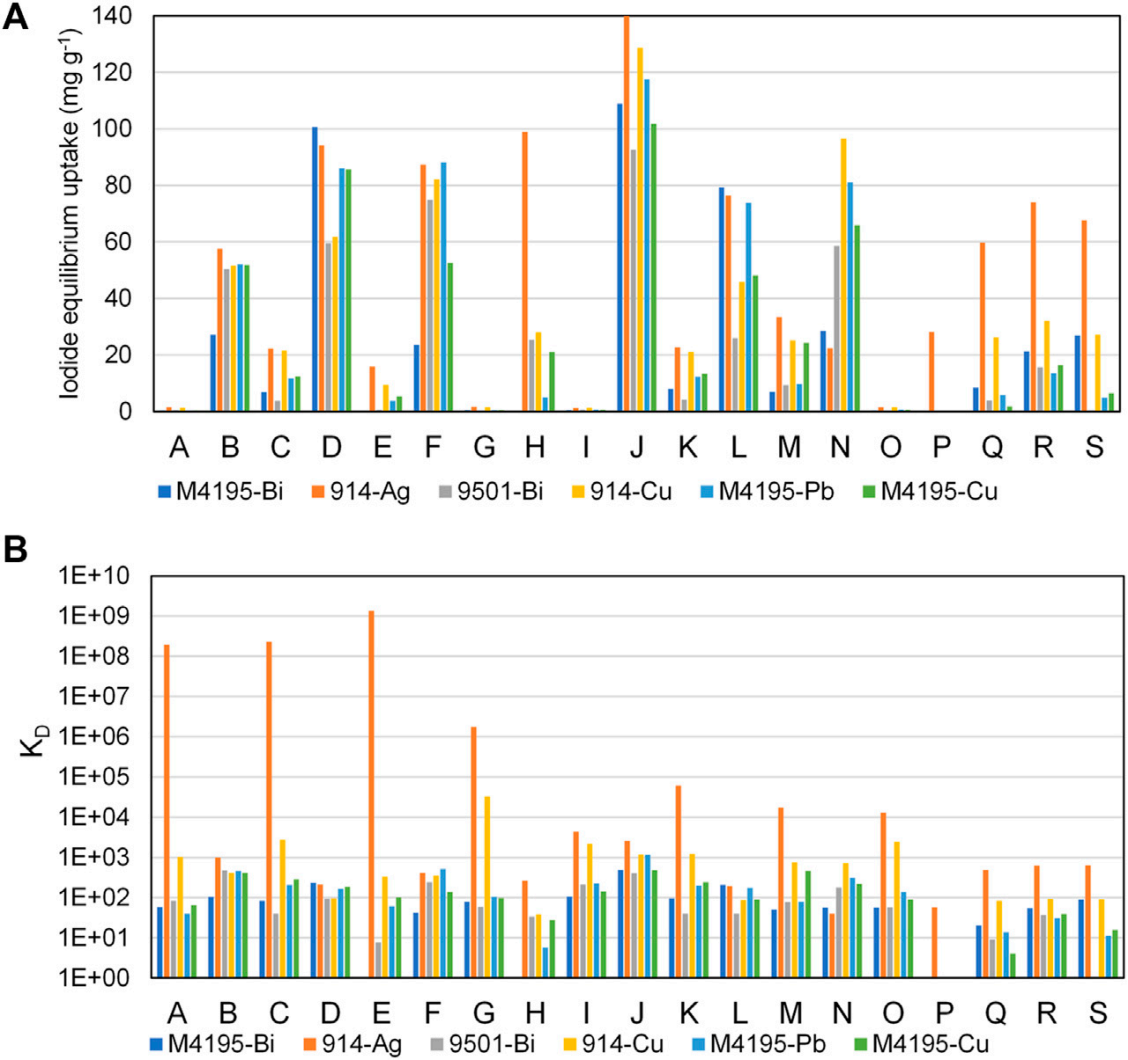
Iodide uptake capabilities of the six overall highest-performing materials, accounting for every sample solution, **(A)** iodide equilibirium uptake (mg·g^−1^) and **(B)** K_D_ (distribution coefficient (Eq. 2)). Experimental parameters are as per Figure 4 and Table 2.

Figure 7 shows an overview of the capabilities of all six progressed functionalities across all sample solutions, both in terms of the recorded uptake (a) and the distribution coefficients (b). The highest performing resin, on both fronts, was S914-Ag. It was the only material that had significant advantages over all others, in terms of pure iodide uptake, in certain solution conditions (Figure 7). Solutions H and P were pH 12, so it is possible that the enhanced S914-Ag uptake in these cases was aided by AgI precipitation. However, no precipitation was observed in the centre-point samples, for which S914-Ag was again clearly superior to its competition (solubility parameters for selected metal halides are given in Supplementary Table S3). The precipitate observed for some Ag-loaded resins appeared to be pale yellowy-grey in colour, which is concurrent with AgI salt, with a degree of Ag (0) contamination, which would be predicted in experiments where light levels were not controlled. Precipitation phenomena may be investigated by XRD measurements (Robshaw et al., 2019a). However, the point behind these experiments was to execute a robust down selection process with a minimum of experimentation and so such measurements were not taken on this occasion.

The success of S914-Ag is due in no small part to the strength of affinity between Ag and iodine. For example, the solubility product (K_sp_) for AgI is 8.52 × 10^−17^, which is five orders of magnitude lower than the equivalent co-contaminant salts, plus AgOH and Ag_2_CO_3_. The same is not true for the equivalent salts of other candidate metals (for example, the K_sp_ for BiI_3_ is 7.71 × 10^−19^, but the parameter for Bi(OH)_3_ is 6.0 × 10^−31^) (Speight, 2016).

With the exception of S914-Ag, it can be seen from Figure 7 that, with certain discrepancies, all of the top six materials responded similarly, in terms of uptake performance, giving a number of viable options of ligand and metal combinations. It is important to note that, within the intended iodine containment process, the efficiency of the adsorbent for aqueous iodine removal is not the only criterion. The compatibility of the spent adsorbent with a suitably inert wasteform, to avoid leaching of radioiodine into the environment is also of huge importance (Riley et al., 2015; Robshaw et al., 2021a) and there are questions to be asked of how the various organic ligands will interact with what will likely be an inorganic wasteform chemistry (Abdel Rahman and Zaki, 2020).

The most dominant factor in predicting high or low iodide uptake appeared to be the initial iodide concentration. This is unsurprising, since the chosen “low” iodide concentration of 10 mg L^−1^ was such that, even if all the iodide in solution had been adsorbed on to the resins, the calculated uptake would still have been significantly less than for most of the solutions with “high” initial iodide concentration. Figure 7B shows that even at low iodide concentration, the affinity of the species for all materials is still considerable across most conditions. This demonstrates the binding strength of the soft acid/soft base interactions which these adsorbents are based on. S914-Ag is notably superior to all other materials in solutions of low iodide concentration (note that the *Y* axis is logarithmic) and would appear to be the logical choice for iodine removal from dilute waste streams. S914-Cu also appears to have superior iodide selectivity to the other candidates. This is likely because the adsorption of Cu^2+^ on to thiourea resins takes place *via* a REDOX interaction and Cu^+^ is a softer acid than Cu^2+^ (which would be the predominant species for M4195-Cu (Bezzina et al., 2019; Robshaw et al., 2020)).

It should be considered that, for the experiments run at low pH, the acidification and presence of light would have changed the iodine speciation, regardless of uptake by the materials, since acidification oxidises the iodide and produces both triiodide and diiodine (Sakurai et al., 1989; Robshaw et al., 2020). Indeed, electrode measurements of many of the acidified solutions of “high” iodide concentration, before adsorbent-contact, showed that the measurable iodide concentration was below 1.0 g L^−1^ (Supplementary Table S2). This was also the case for some of the basic solutions. The most likely explanation is again a shift in speciation (though less significant) towards iodate (Sakurai et al., 1989). The ion-selective electrode measurements would have accurately quantified the removal of iodide only by each adsorbent, but do not yield any useful data with respect to the uptake of other species. The reason total iodine quantification was not attempted was because the intended technology progression is a move to inorganic adsorbent matrices. The majority of triiodide and diiodine present in aqueous solutions associates with ion-exchange resins by partitioning and the formation of charge-transfer complexes with the aromatic ring structures on the resin matrices (Barton et al., 2019). Since this behaviour would not be observed when transitioning from a styrene type matrix to, for example, a silicate matrix, the quantification would not be relevant to the decision-making process. Uptake of iodate by metallated adsorbents has not been studied as widely as that of iodide. It appears to occur by a similar mechanism, but with generally lower affinity (Asmussen et al., 2019). Ultimately, the purpose behind these experiments was to differentiate in the effectiveness for each metal/ligand system for capturing iodide. Nonetheless, it must be acknowledged that the speciation changes during the adsorption equilibrium process add uncertainty to any conclusions drawn.

The reasons for sample solution J providing the best uptake conditions for all materials (in terms of their uptake capacity) are not clear from these data. There appears to be an influence of pH on the competitive co-adsorption of molybdate, since for solution J, molybdate concentration is high and pH is low. Yet, for solutions H and P, molybdate concentration is high and pH is also high and these solutions produced the lowest uptake values of all experiments where iodide concentration was high (Figure 5). This is essentially because when pH is < 7, molybdenum speciation gradually shifts to hexavalent species, which are neutral or cationic (Decamp and Happel, 2013). However, it can also be seen that Solution J yielded rather higher uptake than solution B, with the only differences in solution make-up being that solution B contained no chloride or sulfate. Supplementary Table S2 shows that the measured iodide concentration in solution B was much lower, which is the most likely cause for the performance disparity of many of the materials. From this, it can be inferred that not only does pH impact on iodine speciation, but coexisting contaminants also. This is of relevance, because control over iodine speciation is fundamental to successful removal (Sakurai et al., 1989) It should be noted that neither the group model, nor any individual models found an interaction between pH and molybdate concentration to be significant to iodide removal. It cannot be argued on this basis that simply increasing ionic strength *via* non-competing anions is beneficial to iodide uptake, because the ionic strength of these two solutions was dominated by the 1 M nitrate concentrations. Clearly there are synergistic interactions at work, when certain combinations of species are present, which are too complicated to be predicted and too subtle to be picked up by the statistical analyses used in this case. In the 2_VI_^6-2^ design specification, no main effects are confounded with two-factor interactions, but main effects are confounded with three-factor interactions (Box et al., 1978). It is however noted that the overall group model found that iodide and nitrate interactions were significant and the individual model for S914-Ag found the iodide and molybdate interaction to be significant. We have previously predicted a synergistic co-uptake of iodide and molybdate by Pb-loaded BPA materials (Lambert, 2020) and it has also been reported that adding chloride to a sample solution at a 100:1 M ratio to iodide improved the kinetics of iodide uptake greatly (Zhirnov et al., 1997).

### Performance of the materials compared to previous generations of adsorbents

The uptake capacity data from the first round can be compared to calculated isothermal q_max_ values reported for other adsorbents in the literature field with some confidence. The q_max_ term represents the theoretical saturation capacity of an adsorbent, calculated from a recognised isotherm equation and is widely used in the field of adsorbent development for iodine capture (Robshaw et al., 2021a). In first round experiments, the residual iodide concentration in the sample solutions for every material was >>0 mg L^−1^. Therefore it can be assumed that all adsorbents were close to their maximal capacity. While a relatively crude estimation, this method has been used in relevant literature before (Asmussen et al., 2019). An overview of the uptake capacities of various developed adsorbent classes is shown in Supplementary Table S4. We note that such literature values are calculated on a dry mass basis for the adsorbents, whereas these experimental values were calculated on a hydrated mass basis. Considering the moisture retention data for the resins (Table 1), the calculated q_e_ values can be approximately doubled, for a fairer comparison to the literature. It is noted that the moisture retention values refer to the as-received forms of the resins, rather than the metallated versions. However, the degree of water retention is generally not greatly altered by metallation (Robshaw et al., 2019c; Robshaw et al., 2020). As has been stated, the q_e_ values derived from the first round are of lesser relevance, because of the prevalence of weaker uptake mechanisms thought to be occurring (Singare, 2014; Barton et al., 2019). Nonetheless, it can be seen that the most capacious adsorbents removed more iodine from solution that any class of adsorbent (with the exception of other ion-exchange resins) (Lambert, 2020; Robshaw et al., 2020).

Even with the extreme aqueous conditions studied in the second experimental round, the uptake abilities of the top six materials compare favourably to most metallated adsorbents reported in the literature. These candidates all exhibited a mean iodide uptake of >20 mg g^−1^ across all sample solutions (Figure 6), equating to >40 mg g^−1^ on a dry mass basis, and this comfortably exceeds the maximal uptake values for materials such as Ag-loaded zeolites (20 mg g^−1^) (Tauanov and Inglezakis, 2019), Ag-loaded activated carbon (13 mg g^−1^) (Karanfil et al., 2005) and copper oxides adsorbents (23 mg g^−1^) (Mao et al., 2017), which were recorded in ideal conditions (iodide only). It does however remain to be seen whether the capacity of the future inorganic materials, albeit with equivalent functionalities, can match that of their resin equivalents, with preliminary data suggesting this is unlikely (Lambert, 2020; Robshaw et al., 2021b). Other inorganic adsorbents previously reported have exhibited iodide capacities >100 mg g^−1^, though again, in ideal conditions (Liu et al., 2016; Chen et al., 2018). Where more challenging uptake conditions have been introduced in the literature, this has been done in the form of large excesses of single competing anions (Liu et al., 2015; Zhang et al., 2017) or an accurate simulation of an industrial wastestream (Asmussen et al., 2016; Asmussen et al., 2019), neither being a meaningful comparison to our own results.

Further work is also required to determine the extent of metal-leaching from the six progressed materials at extreme pH values, which is important, because of the need to avoid leaching of heavy and/or toxic metals from adsorbent columns, during dynamic operations. This has not yet been checked for any of these materials, with the exception of M4195-Cu and M4195-Pb from previous publications. These suggested that significant leaching would occur for Cu at pH 0 and 12, and for Pb at pH 0 (Lambert, 2020; Robshaw et al., 2020).

Overall, the fractional factorial design approach has succeeded in the goal of narrowing down the list of feasible adsorbents to a sensible number, using efficient methodology. There has also been partial success in the goal of identifying solution chemistry that is advantageous and detrimental to iodide uptake. For example, it is strongly predicted that the competing effect of molybdate on iodide removal can be avoided by acidification of the solution. It is also shown that the better-performing adsorbents are very resistant, in terms of uptake performance, to extreme pH.

## Conclusion

A total of 32 different metallated ion-exchange resins, comprised of eight commercial resin matrices and four loading metals, have been assessed for their capabilities for removal of iodide ions from aqueous solution. The assessment was run over two experimental stages. The first was performed with solutions of pure NaI and was used to eliminate materials that did not function according to the predicted mechanism of ligand-exchange, with formation of metal iodide complexes upon the resin surface. The second stage introduced relevant industrial conditions, using varying pH, iodide concentration and concentrations of competing anions. To increase the efficiency of the down selection process, a fractional factorial approach was used. A group model was constructed from the data, to account for the effect of solution chemistry parameters on the iodide uptake capabilities of the resins. Discounting the materials that returned a large number of 0 uptake values, the model fit the whole dataset with a *R* ^2^ of 59%, finding that iodide concentration, nitrate concentration, pH and two-way interactions between them were significant. Individual models were also created for each material, which had an *R* ^2^ range of 18–82%. They mainly found that across all models, only iodide concentration was a significant parameter, which is consistent with calculations for the uptake capacity of an adsorbent. This suggests that the best-performing materials were, in general, resistant to the challenging chemical conditions studied. There were also a number of postulated unexpected chemical interactions, possibly involving changes to iodine speciation and/or synergistic co-uptake with other contaminants, which were too subtle to be found statistically significant. Nonetheless, the second experimental stage succeeded in further dividing high-and low-performing adsorbents. Six candidates were chosen to progress to further project stages. The silver-loaded thiourea functionality S914-Ag was seemingly the most promising material tested. However, all candidates merit further investigation, with respect to conversion of the adsorbent matrix to an inorganic form, further testing with a fully-representative nuclear aqueous wastestream and pathways to wasteform conversion.

## Data Availability

The original contributions presented in the study are included in the article/Supplementary Material, further inquiries can be directed to the corresponding author.
