# Supplementary material for: Functionality screening to help design effective materials for radioiodine abatement
Source: Front Chem. 2022 Oct 18;10:997147. doi: 10.3389/fchem.2022.997147 (PMC9623042; doi:10.3389/fchem.2022.997147)
Supplement: Supplementary file 1 [file DataSheet1.docx]

Supplementary Material

# Data Visualisation

**Figure S1** shows histograms of the iodide uptake for each material in 10 mg/g bins.


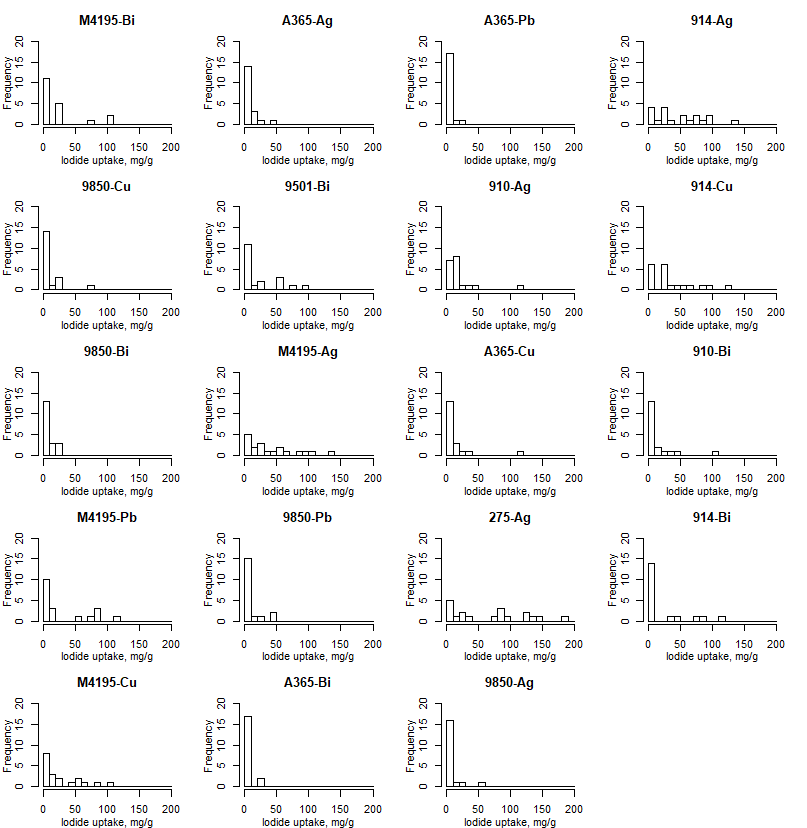


***Figure S1****. Histograms of the measured iodide uptake values for each material*

Note that several of the materials had multiple 0 values that will all be shown in the first bar of the histogram. From these we can see that, for example, A365-Pb, 9850-Bi and A365-Bi all have very low iodide uptake across the whole range of input parameters tested and these would likely not be good candidates for further testing.

Figure S2 shows boxplots (minimum, lower quartile, median, upper quartile and maximum) of the three centre point values for each parameter. Centre points, spread throughout the randomised design, are used to understand between-experiment variability and ideally would produce very similar results. Generally, the materials have quite consistent centre points though some stand out as more variable. In some cases, such as M4195-Ag, the boxplot is symmetric showing the centre points are evenly spread out. And in other cases, such as 9850-Cu, the box plot is skewed showing that two of the centre points agree quite closely compared to the other.


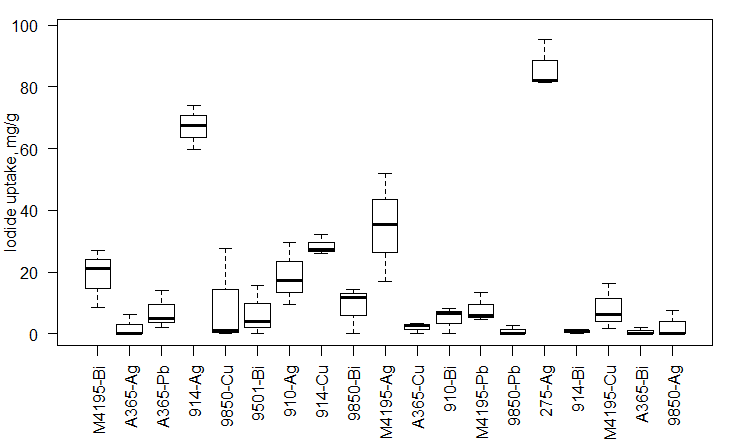


***Figure S2****. Boxplots of centre point values.*

The residual plots are shown in Figure S3. The residual vs fitted plots generally shows a good random scatter. The straight line of points that decreases as fitted values increases are mostly data points with a 0 iodide uptake measurement, and hence the model does not fit these points well. This is the same for the scale-location plot. The q-q plot shows a good fit to the normal line, with the exception in the bottom tail for the same outliers as the residual plot. The residual vs leverage plot does not highlight any influential points. It is also possible to look at the fit by material using the residual plot. This is shown in Figure S4. None of the materials stand out as having a poorer fit overall, as would be expected from the modelling looking for the best fit to the group data.


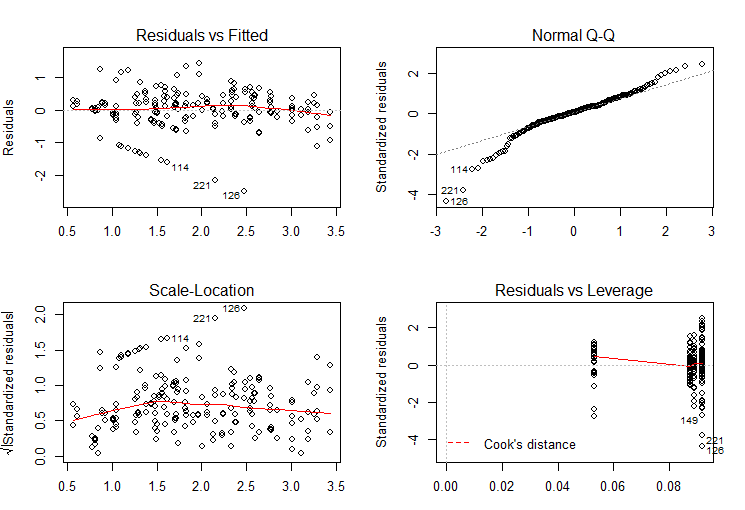


***Figure S3****. Residual plots for the group linear model.*


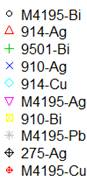

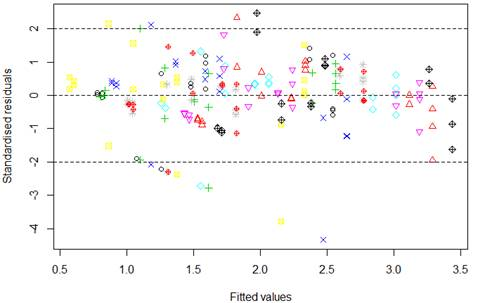


***Figure S4****. Standardised residuals for the group model, coloured by material.*

The fitted model can be used to make predictions across the range of input parameters and these are used to make contour plots to visualise the areas for best iodide uptake. These are shown for the 10 materials included in Figures S5-14, where iodide and nitrate are varied continuously on the x and y axes respectively, pH is varied at 3 levels, one for each plot, and predicted iodide uptake is shown in the contours in 5 mg/g intervals. Using the contour plots, it is easier to see the interactions between the iodine, nitrate and pH variables with the shape of the contours and understand the combinations that give the best iodide uptake.


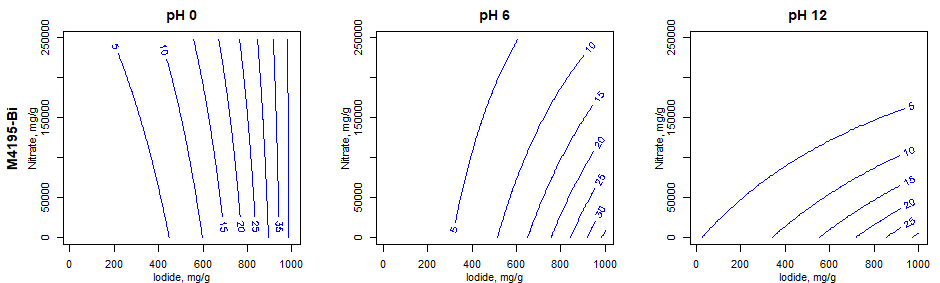


***Figure S5****. Predicted contour plots for M4195-Bi from the group model.*


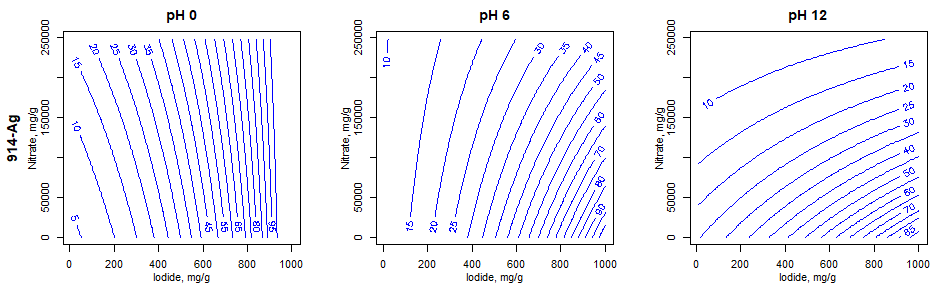


***Figure S6****. Predicted contour plots for 914-Ag from the group model.*


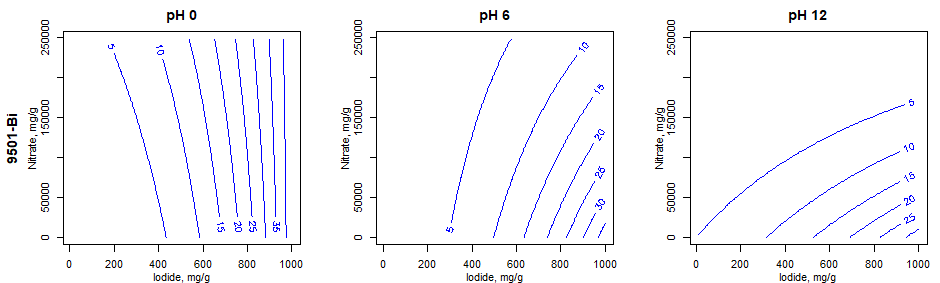


***Figure S7****. Predicted contour plots for 9501-Bi from the group model.*


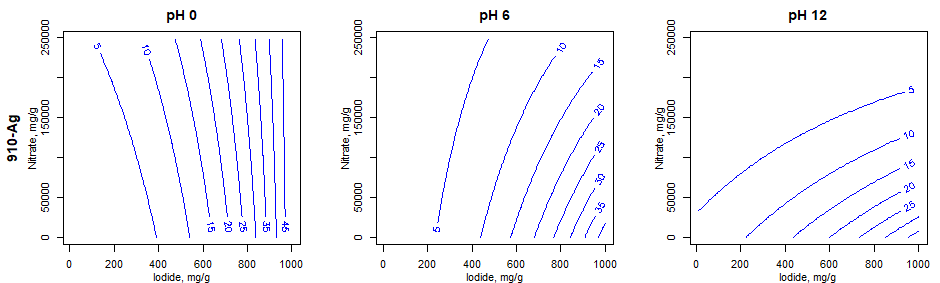


***Figure S8****. Predicted contour plots for 910-Ag from the group model.*


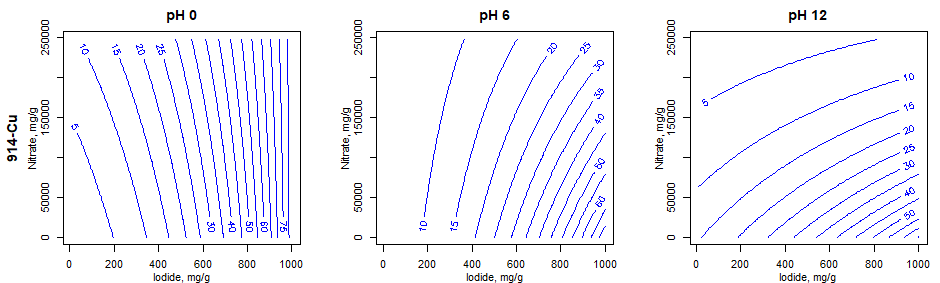


***Figure S9****. Predicted contour plots for 914-Cu from the group model.*


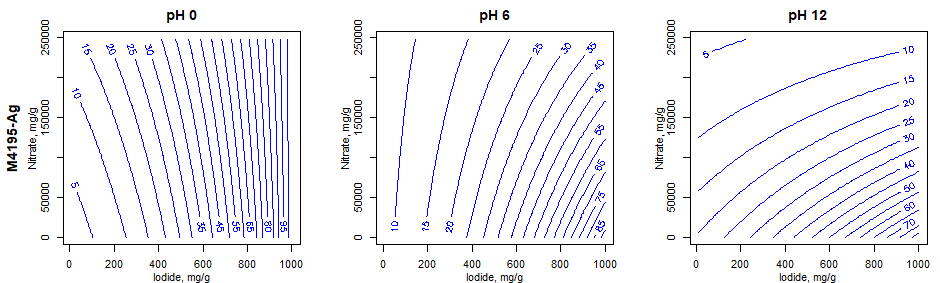


***Figure S10****. Predicted contour plots for M4195-Ag from the group model.*


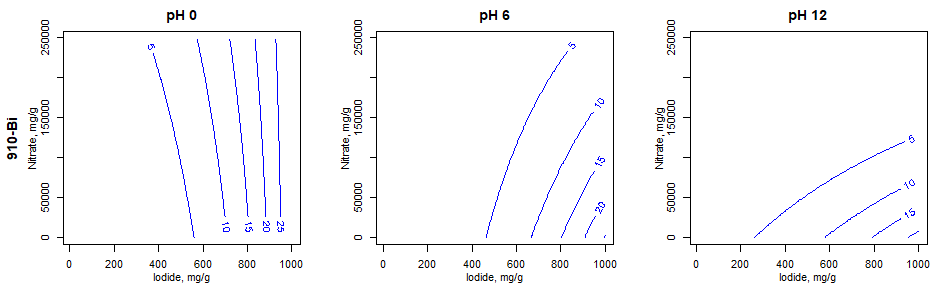


***Figure S11****. Predicted contour plots for 910-Bi from the group model.*


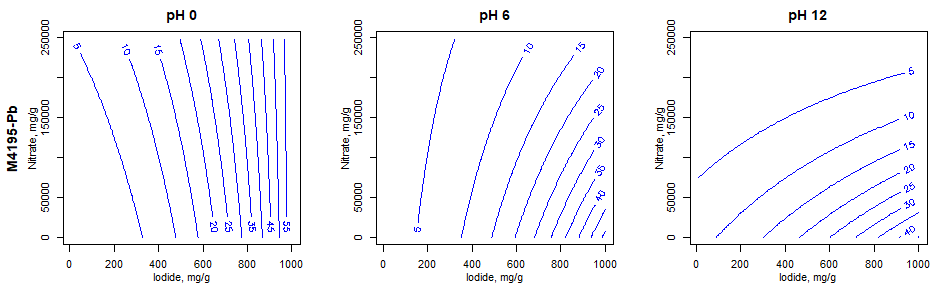


***Figure S12****. Predicted contour plots for M4195-Pb from the group model.*


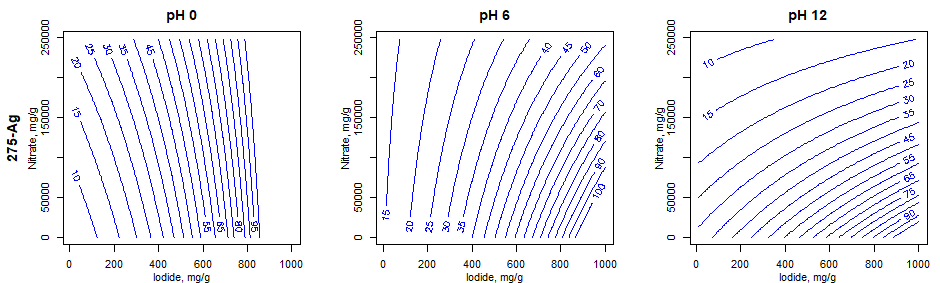


***Figure S13****. Predicted contour plots for 275-Ag from the group model.*


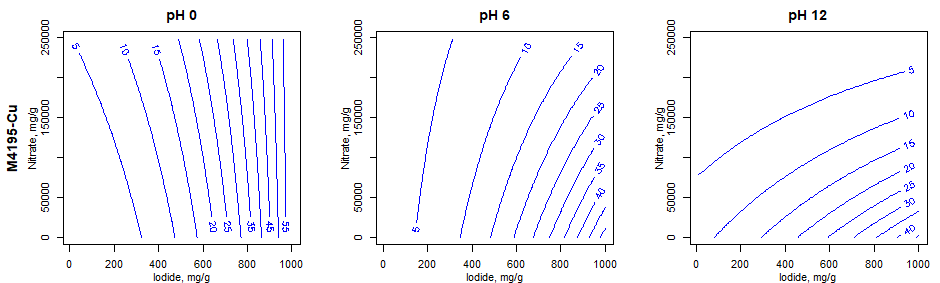


***Figure S14****. Predicted contour plots for M4195-Cu from the group model.*

**2 Individual materials models (with S914-Ag as an example)**

It is also possible to build models for each individual material to explore what effects the parameters have on the iodide uptake. The individual model for 914-Ag (using a fourth root transformation) finds iodide and molybdate to be significant, including the interaction between the two. This is different from the combined model as nitrate and pH are not found to be significant and molybdate is. The model is given below.

${uptake}^{\frac{1}{4}} = 1.18 + 1.74\times{10}^{-3}\times\text{iodide} + 6.84\times{10}^{-4}\times molybdate - 6.30\times{10}^{-7}\times\text{iodide}\times\text{molybdate}$ (Eqn. S1)

The R^2^ for this model is 74% which shows a good amount of the variation in the data in explained by the model. In the residual plots (Figure S15) two points stand out as potential outliers, identified as points 71 and 73. Further investigation into these points show that they have the higher input iodide but the two lowest iodide uptake values, hence a poorer fit. Figure S16 shows the contour plots based on the prediction from this model over the ranges of iodide and molybdate. Figure S17 shows a comparison between the experimental data and the group and individual model predictions. Overall, the two models predict quite similar values.


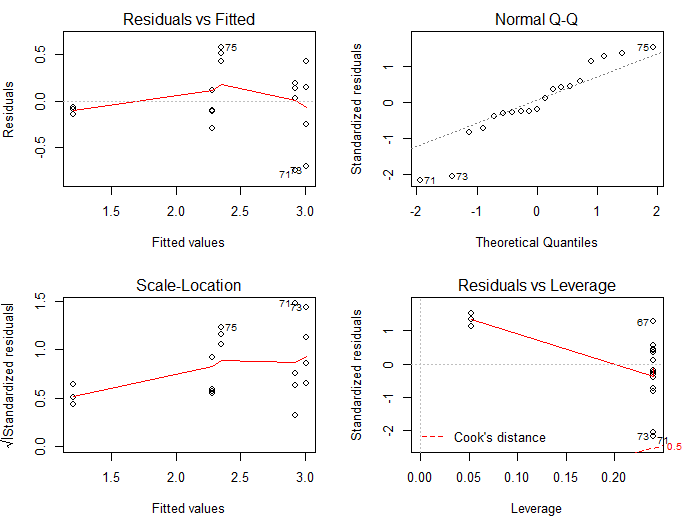


***Figure S15.*** *Residual plots for the individual model for 914-Ag.*


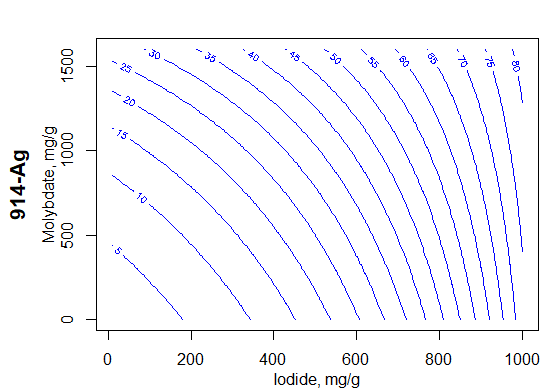


***Figure S16****. Contour plots from the individual model for 914-Ag.*


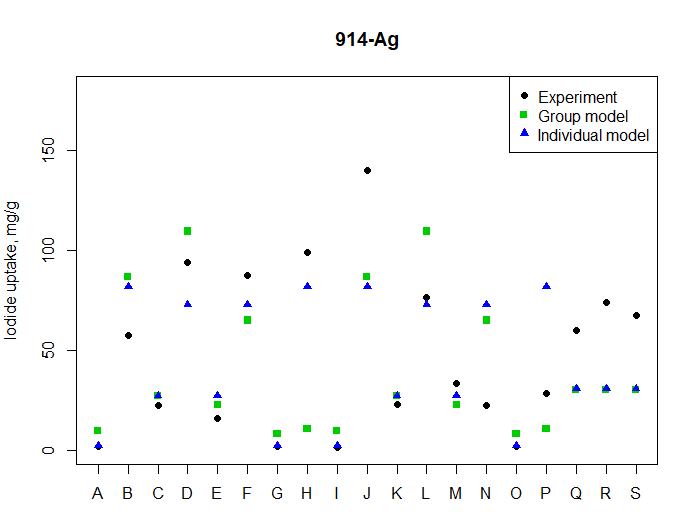


***Figure S17****. Prediction comparisons between the group model and the individual model for 914-Ag.*

**3 General comments on individual models**

Some of the individual models were quite poor fits (low R^2^ values) and with only one or two parameters found to be significant there is a lot of variation not being picked up. The correlation between pH and nitrate should be considered here – even when the model did not find the individual terms (main effects) to be significant, the interaction may still be.

Due to the confounding issue explained previously, we could not use the preferred backwards step-wise approach and the forward approach was used instead. This interaction term was added after the final step-wise model was found to explore the effect. The interaction between pH and nitrate was found to be significant, and improve the overall model fit, for 914-Ag, 914-Cu, M4195-Ag, M4195-Pb and M4195-Cu. Note that none of the parameters were found to be significant in the model for 910-Ag, as could be expected by the small variation in iodide uptake values (Figure S2). Most materials found iodide to be significant. The pH, nitrate and molybdate parameters were also found to be significant for some individual materials.

**Table S1**. Significant parameters for the individual models of each of the 10 materials included in the group model, with their corresponding R^2^ values. Green = significant. Red = not significant.

| **Material** | **Iodide** | **pH** | **Nitrate** | **Chloride** | **Molybdate** | **Sulfate** | **R^2^** |
| --- | --- | --- | --- | --- | --- | --- | --- |
| M4195-Bi |  |  |  |  |  |  | 44% |
| 914-Ag |  |  |  |  |  |  | 82%^†^* |
| 9501-Bi |  |  |  |  |  |  | 33% |
| 910-Ag |  |  |  |  |  |  | - |
| 914-Cu |  |  |  |  |  |  | 49%* |
| M4195-Ag |  |  |  |  |  |  | 80%* |
| 910-Bi |  |  |  |  |  |  | 18% |
| M4195-Pb |  |  |  |  |  |  | 65%* |
| 275-Ag |  |  |  |  |  |  | 72% |
| M4195-Cu |  |  |  |  |  |  | 55%* |

^†^ Iodide:Molybdate interaction significant, * pH:nitrate interaction significant

**Table S2.** The initial iodide concentrations of all sample solutions, as measured by ISE. In all cases, the mass of NaI added to the total solution volume was such as to give an intended concentration of 1.00 g·L^-1^.

| **Solution reference** | **pH** | **Intended iodide concentration**  **(mg·L^-1^)** | **Measured iodide concentration**  **(mg·L^-1^)** | **Difference**  **(mg·L^-1^)** |
| --- | --- | --- | --- | --- |
| B | 0 | 1,000 | 402 ± 28 | 598 |
| D | 12 | 1,000 | 978 ± 10 | 22 |
| F | 0 | 1,000 | 692 ± 3 | 307 |
| H | 12 | 1,000 | 884 ± 6 | 116 |
| J | 0 | 1,000 | 781 ± 10 | 219 |
| L | 12 | 1,000 | 791 ± 5 | 209 |
| N | 0 | 1,000 | 677 ± 4 | 323 |
| P | 12 | 1,000 | 656 ± 17 | 344 |
| Q | 6 | 505 | 456 ± 2 | 44 |
| R | 6 | 505 | 511 ± 4 | 11 |
| S | 6 | 505 | 449 ± 2 | 51 |

**Table S3.** Selected metal iodide solubility parameters (25 °C) of the various metals explored in the functionalised materials [1].

| **Name** | **Formula** | **K_SP_** | **Log_10_ K_SP_** |
| --- | --- | --- | --- |
| Bismuth iodide | BiI_3_ | 7.71 x 10^-19^ | -18.11 |
| Copper (I) iodide | CuI | 1.27 x 10-12 | -11.90 |
| Lead (II) iodide | PbI_2_ | 9.8 x 10^-9^ | -8.01 |
| Silver(I) iodide | AgI | 8.52 x10^-17^ | -16.07 |

**Table S4.** Condensed summary of adsorption capabilities of various metal-functionalised materials in the field of iodide-capture.

| **Adsorbent type** | **Literature values range** | | | | **References** |
| --- | --- | --- | --- | --- | --- |
|  | **Functionalising metals used** | **Iodide capacity** | | **Equilibrium time**  **(hr)** |  |
|  |  | **(mmol g^-1^)** | **(mg g^-1^)** |  |  |
| Activated carbon | Ag, Cu | 0.10-0.32 | 13-41 | 1-72 | [2, 3] |
| Ion-exchange resin | Ag, Cu, Pb | 0.20-3.5 | 26-450 | <1-2.5 | [4-6] |
| Zeolite | Ag, | 0.029-0.16 | 3.7-20 | 24-240 | [7-9] |
| TiO_2_ | Ag, | 1.7-3.4 | 210-430 | 1-6 | [10, 11] |
| SiO_2_ | Ag, | 0.69 | 88 | 1 | [12] |
| Other mineral | Ag, Bi, Cu, Pb | 0.18-2.9 | 23-370 | <1-360 | [6, 13-15] |

**4 Supporting information references**

[1] C. Balarew, E. V. Zagnit’ko, J. Eysseltova, and J.-J. Counioux, “IUPAC-NIST Solubility Data Series. 85. Transition and 12–14 Main Group Metals, Lanthanide, Actinide, and Ammonium Halates,” Journal of physical and chemical reference data, vol. 37, no. 2, pp. 933–1118, 2008, doi: 10.1063/1.2804088.

[2] T. Karanfil, E.C. Moro, S.M. Serkiz, Environmental Technology, 26 (2005) 1255-1262.

[3] X.Y. Zhang, P. Gu, X.Y. Li, G.H. Zhang, Chemical Engineering Journal, 322 (2017) 129-139.

[4] C. Decamp, S. Happel, Journal of Radioanalytical and Nuclear Chemistry, 298 (2013) 763-767.

[5] T.J. Robshaw, S.M. Griffiths, A. Canner, J.P. Bezzina, D.B. Hammond, S. Van Meurs, M.D. Ogden, Chemical Engineering Journal, 390 (2020) 124647-124659.

[6] F. Lambert, University of Sheffield, (2020).

[7] R.M. Asmussen, J.J. Neeway, A.R. Lawter, A. Wilson, N.P. Qafoku, Radiochimica Acta, 104 (2016) 905-913.

[8] J. Warchol, P. Misaelides, R. Petrus, D. Zamboulis, Journal of Hazardous Materials, 137 (2006) 1410-1416.

[9] Z. Tauanov, V.J. Inglezakis, Science of the Total Environment, 682 (2019) 259-270.

[10] A. Bo, S. Sarina, Z.F. Zheng, D.J. Yang, H.W. Liu, H.Y. Zhu, Journal of Hazardous Materials, 246 (2013) 199-205.

[11] S.S. Liu, N. Wang, Y.C. Zhang, Y.R. Li, Z. Han, P. Na, Journal of Hazardous Materials, 284 (2015) 171-181.

[12] R.M. Asmussen, J. Matyas, N.P. Qafoku, A.A. Kruger, Journal of Hazardous Materials, 379 (2019).

[13] P. Mao, Y. Liu, Y. Jiao, S.W. Chen, Y. Yang, Chemosphere, 164 (2016) 396-403.

[14] P. Mao, L.Y. Qi, X.D. Liu, Y. Liu, Y. Jiao, S.W. Chen, Y. Yang, Journal of Hazardous Materials, 328 (2017) 21-28.

[15] Y.Y. Chen, S.H. Yu, Q.Z. Yao, S.Q. Fu, G.T. Zhou, Journal of Colloid and Interface Science, 510 (2018) 280-291.
